# Supplementary material for: The role of religious narratives and religious orientation towards concerns for the natural environment and animal welfare
Source: PLoS One. 2022 Aug 11;17(8):e0271515. doi: 10.1371/journal.pone.0271515 (PMC9371258; doi:10.1371/journal.pone.0271515)
Supplement: S1 File — (DOCX) [file pone.0271515.s001.docx]

# English Version

## Stewardship narrative (SN)

**MAN AND NATURE**

Human relationship with the nature, with their fellow human and with Allah SWT has been set in Islam. Habluminallah tells the human relationship with Allah SWT through human obedience to all His commands and prohibitions. Habluminannas is a human relationship with humans, and Habluminal'alam, tells the preservation of the nature.

The Qur'an explains the importance of nature. Of the total 57 words that mention 'Jannah' (paradise), 45 of them come from the word 'Jannah' which can be interpreted as 'forest'. The beauty of Heaven is described as "green forests, densely filled with beautiful fruits and flowing streams of clear and cool streams". In addition, many verses in the Qur'an and hadith that explain the importance of keeping (planting) trees with great rewards for those who run them:

"If the Apocalypse is about to happen, while among you there is the seeds of palm then if he is able to plant before doomsday then let him plant it." (Hadith Ahmad's History)

"It is not a Muslim planting a tree, nor planting crops, then the tree or plant is eaten by birds, humans or animals but becomes alms"

(Hadith Imam Bukhari and Muslim narrated by Anas bin Malik)

"It is not a Muslim planting a crop unless it is eaten from him a charity, what is stolen from him is alms, what the wild animal eats is alms, which is eaten by birds is alms, and what others take is also alms in another pronunciation alms till the end of the world "

(Muslim hadith narrated by Jabir bin Abdullah)

The Prophet's Word, "Who planted a tree, will be rewarded with as much as a tree and its fruit" (narrated by Abu Ya'la)

Even forbidden to cut Timber of war time:

 "Whoever kills a child, cuts down a fruitful tree, kills animals for his skin, kills a farmer or a parent who does not go to war, then he will not return in a state of affluence"

(Hadith narrated Ahmad bin Hambal)

In Islam, the destruction of the environment or nature can be categorized as the act of hirobah (action of war). Muslims are asked to preserve the environment with the reward of planting trees and keeping nature / environment as a practice that never breaks up until the end of time (the hereafter).

## Human dominance narrative (DN)

**HUMAN AS THE NOBLE BEING**

Man was created by Allah SWT as the most noble creature among the other creatures and as the best creature (*insanul kamil*).

"We created man in the best possible form." (At-Tin: 4)

Allah Almighty has explained the meaning of the noble man on earth with perfection over other beings on the gift of God (man is given the best knowledge, reason and form which is not given to His other creatures)

"And verily We have honored the children of Adam, We transport them on land and in the seas, We give them blessing from the good and We exalt them with perfect advantages over most of the creatures we have created." (Al-Israa ': 70)

Shaykh Abdurrahman bin Nasir As-Sa'diy Rahimakumullah explained,

"Because God has specialized in man in the form of a position and a virtue not found in any other creature" (Tafsir Karimirahman p.333, Muassasah Risalah, cet, I, 1420 H, Asy Syamilah).

Through the dialogue between the angels and Allah SWT about the creation of Adam (in Surah Baqarah verses 30 to 33 and Surah verses 71-72), Allah explains the reason why He chose man as Caliph and gave him the highest position of all beings. This extraordinary position of human beings is also found in various parts of the Qur'an. Some of them are as follows:

"And He bowed the night and the day, the sun and the moon for you. And the stars are subdued (for you) by His command. Verily in that are Signs for those who understand, "(An-Nahl 12)

"And He (subdued also) what He created for you on this earth in various ways. Verily in that is a Sign (the power of Allah) for those who take the lesson. "(An-Nahl 13)

"And it is He who hath subdued the sea (for you), that ye may eat of it fresh flesh (fish), and ye remove from the sea the jewels which ye wear; and ye shall see the ark sail unto him, and that ye seek (profit) from his bounty, and that ye may give thanks. "(An-Nahl 14)

In order not to misinterpret the human being as the most noble being, it is worth explaining that human power is also limited. Secondly, the power given to man is not absolute over everything. Because the absolute and the powerful are back again to Allah Almighty, in accordance with the nature of Allah which is Al-Qadir (All-Wise), Al-Muqtadir (Powerful), Malik-ul-Mulk (Supreme Ruler of the Kingdom (Universe), Al-Khaliq ( The Creator).

# Indonesia Version

## Stewardship narrative (SN)

**MANUSIA DAN ALAM**

Hubungan manusia dengan lingkungan, sesama dan dengan Allah SWT telah diatur dalam islam. Habluminallah menceritakan hubungan manusia dengan Allah SWT melalui ketaatan manusia kepada semua perintah dan laranganNya. Habluminannas adalah hubungan manusia dengan manusia, dan Habluminal ‘alam, menceritakan kelestarian lingkungan dan alam.

Al-Quran banyak menjelaskan pentingnya alam. Dari total 57 kata yang menyebutkan ‘Jannah’ (surga), 45 diantaranya berasal dari kata ‘Jannah’ yang bisa diartikan sebagai ‘hutan’. Keindahan Surga digambarkan dengan “hutan-hutan yang hijau, lebat penuh dengan buah-buahan bangunan yang asri dan mengalirnya sungai-sungai yang jernih dan sejuk”. Selain itu, banyak ayat di Al-Quran dan hadits yang menjelaskan pentingnya menjaga (menanam) pohon dengan pahala yang besar bagi mereka yang menjalankannya:

“Sekiranya kiamat hendak terjadi, sedangkan diantara kalian ada bibit kurma maka apabila dia mampu menanam sebelum terjadi kiamat maka hendaklah dia menanamnya.” (Hadits Riwayat Ahmad)

“Tidaklah seorang muslim menanam pohon, tidak pula menanam tanaman, kemudian pohon atau tanaman tersebut dimakan oleh burung , manusia atau binatang melainkan menjadi sedekah”

(Hadits imam Bukhari dan Muslim diriwayatkan oleh Anas bin Malik)

“Tidaklah seorang muslim menanam tanaman kecuali yang dimakan darinya merupakan sedekah, apa yang dicuri darinya merupakan sedekah, apa yang dimakan oleh binatang buas merupakan sedekah, yang dimakan oleh burung merupakan sedekah, dan apa yang diambil oleh orang lain juga merupakan sedekah dalam lafal lain merupakan sedekah sampai akhir kiamat”

(Hadits Muslim yang diriwayatkan Jabir bin Abdullah)

Sabda Rasul SAW, “Siapa yang menanam pohon, niscaya diberikan pahala sebanyak pohon dan buahnya” (diriwayatkan oleh Abu Ya’la)

Bahkan dilarang menebang Pohon waktu perang:

“Barang siapa membunuh anak kecil, menebang pohon berbuah, membunuh hewan untuk di ambil kulitnya, membunuh petani atau orang tua yang tidak ikut berperang,maka dia tidak akan kembali dalam keadaan berkecukupan”

(Hadits riwayat Ahmad bin hambal)

Dalam Islam, perusakan lingkungan atau alam dapat dikatagorikan sebagi tindakan hirobah (tindakan perang). Umat muslim diminta untuk menjaga lingkungan dengan pahala menanam pohon dan mejaga alam/lingkungannya sebagai amalan yang tak pernah putus sampai akhir jaman (dunia akhirat).

## Human dominance narrative (DN)

**MANUSIA MAKHLUK MULIA**

Manusia diciptakan oleh Allah SWT sebagai makhluk yang paling mulia diantara makhluk-makhluk lain dan sebagai makhluk terbaik (insanul kamil).

"Sesungguhnya Kami telah menciptakan manusia dalam bentuk yang sebaik-baiknya”. (At-Tin: 4)

Allah SWT telah menjelaskan arti dari mulianya manusia di muka bumi dengan kesempurnaan melebihi makhluk yang lain atas karunia dari Allah (manusia diberikan pengetahuan, akal dan bentuk yang paling baik yang tidak diberikan pada makhluk ciptaanNya yang lain)

“Dan sesungguhnya telah Kami muliakan anak-anak Adam, Kami angkut mereka di daratan dan di lautan , Kami beri mereka rizki dari yang baik-baik dan **Kami lebihkan mereka dengan kelebihan yang sempurna atas kebanyakan makhluk yang telah Kami ciptakan.”** (Al-Israa’ : 70)

Syaikh Abdurrahman bin Nashir As-Sa’diy Rahimakumullah menjelaskan,

“Karena Allah telah mengkhususkan manusia berupa kedudukan dan keutamaan yang tidak ada pada makhluk lainnya” (Tafsir Karimirahman hal. 463, Muassasah Risalah, cet, I, 1420 H, Asy Syamilah).

Melalui dialog antara para malaikat dengan Allah SWT tentang penciptaan Nabi Adam As (dalam surat Al Baqarah ayat 30 sampai 33 dan surat Shad ayat 71-72), Allah SWT menjelaskan alasan mengapa Ia memilih manusia sebagai khalifah dan memberikannya kedudukan yang tinggi dari semua makhluk. Posisi dan kelebihan-kelebihan manusia yang istimewa ini juga banyak ditemukan di berbagai bagian di dalam Al-Quran. Beberapa diantaranya adalah sebagai berikut:

*“Dan Dia menundukkan malam dan siang, matahari dan bulan untukmu. Dan bintang-bintang itu ditundukkan (untukmu) dengan perintah-Nya. Sesungguhnya pada yang demikian itu benar-benar ada tanda-tanda (kekuasaan Allah) bagi kaum yang memahami(nya),” (An-Nahl 12)*

*“dan Dia (menundukkan pula) apa yang Dia ciptakan untuk kamu di bumi ini dengan berlain-lainan macamnya. Sesungguhnya pada yang demikian itu benar-benar terdapat tanda (kekuasaan Allah) bagi kaum yang mengambil pelajaran.” (An-Nahl 13)*

*“Dan Dialah, Allah yang menundukkan lautan (untukmu), agar kamu dapat memakan daripadanya daging yang segar (ikan), dan kamu mengeluarkan dari lautan itu perhiasan yang kamu pakai; dan kamu melihat bahtera berlayar padanya, dan supaya kamu mencari (keuntungan) dari karunia-Nya, dan supaya kamu bersyukur.” (An-Nahl 14)*

Agar tidak salah menafsirkan manusia sebagai mahluk yang paling mulia maka layak untuk dijelaskan bahwasanya kekuasaan manusia juga terbatas. Yang kedua, kekuasaan yang diberikan kepada manusia tidak mutlak atas segalanya. Karena yang mutlak dan berkuasa adalah kembali lagi kepada Allah SWT, sesuai dengan sifat Allah yang *Al-Qadir* (Maha Berkehendak), *Al-Muqtadir* (Maha Berkuasa), *Malik-ul-Mulk* (Maha Penguasa Kerajaan (Semesta), *Al-Khaliq* (Maha Pencipta).
