# Supplementary material for: The role of religious narratives and religious orientation towards concerns for the natural environment and animal welfare
Source: PLoS One. 2022 Aug 11;17(8):e0271515. doi: 10.1371/journal.pone.0271515 (PMC9371258; doi:10.1371/journal.pone.0271515)
Supplement: S3 File — (DOCX) [file pone.0271515.s003.docx]

# Appendix 2: Questionnaires translated to Bahasa Indonesia

## **Kontrol Data**

Isilah pertanyaan-pertanyaan di bawah ini.

Jika pertanyaan berbentuk pilihan, lingkari jawaban yang sesuai dengan anda.

Jika pertanyaan berbentuk isian maka isilah dengan singkat

| 1. Jenis kelamin: **Perempuan/Laki-laki** (lingkari salah satu) | |
| --- | --- |
| 1. Apakah anda anggota ATAU penyumbang organisasi/ormas keagamaan? **Ya/Tidak** (lingkari salah satu) | |
| ***Jika anda menjawab “ya”***, Bergerak di bidang apa organisasi anda? (bisa melingkari lebih dari satu) | |
| - 1. Dakwah   2. Sumbangan/derma   3. Politik   4. Jurnalistik Berita/Penggiat Media Sosial | - 1. Yayasan/Lembaga Sosial Masyarakat (LSM) kemanusiaan   2. Peningkatan ekonomi (contoh: arisan, koperasi, dll)   3. Lain-lain: __________________________ (tuliskan) |
| 1. Di sekolah mana anda bekerja: (isi dengan singkat nama sekolah dan domisili sekolah)   _____________________________________________________________________________________ | |
| 1. Tahun berapa anda lahir? ____________ (tulis keseluruhan 4 angka, contoh: 1989) | |
| 1. Dimana anda tinggal?   Propinsi: ___________________________________, Kabupaten: _______________________________ | |
| 1. Jenjang pendidikan yang telah ditamatkan? (lingkari salah satu di bawah ini) | |
| - 1. Tidak sekolah   2. SD/Madrasah Ibtidayah   3. SMP/Madrasah Tsanawiyah   4. SMU/Madrasah Aliyah | - 1. Diploma D1/D3   2. Strata Pertama (S1)   3. S2/S3   4. Lain-lain: ________________ (tuliskan) |
| 1. Apakah anda anggota atau penyumbang organisasi sosial yang bergerak di bidang di bawah ini: | |
| - 1. Peningkatan kesejahteraan binatang **Ya/Tidak**   2. Pelestarian lingkungan hidup/alam **Ya/Tidak** | - 1. Peningkatan kesehatan/hak asasi manusia **Ya/Tidak** |
| 1. A. Bagaimana status pernikahan anda? (lingkari salah satu di bawah ini) | |
| - 1. Lajang/belum menikah   2. Menikah | - 1. Janda/Duda |
| B. Apakah anda mempunyai anak? **Ya/Tidak** (lingkari salah satu) | |
| 1. Didaerah apa anda bermukim? | |
| - 1. Daerah Perkotaan (termasuk kota kecil)   2. Di luar kawasan kota seperti Desa/Dusun | |
| 1. Jenis tempat tinggal anda? (lingkari salah satu di bawah ini) | |
| - 1. Apartment   2. Kost-kostan/Kontrakan | - 1. Rumah sendiri   2. Rumah bersama orang tua |
| 1. Apakah anda mempunyai kebun/taman? **Ya/Tidak** (lingkari salah satu) | |
| 1. Apa pekerjaan utama anda? | |
| - 1. Wiraswasta   2. Pegawai Swasta   3. Pegawai Negeri Sipil (PNS)   4. Pensiun   5. Pelajar/Beasiswa | - 1. Pekerja sosial   2. Peneliti/dosen   3. Bertani/beternak   4. Tidak ingin menjawab   5. Lain-lain: ________________ (tuliskan) |
| 1. A. Apakah Agama anda? __________________________ (boleh tidak menjawab) | |
| B. Suku anda: __________________________ (boleh tidak menjawab) | |
| C. Apakah Agama/Spiritualitas penting dalam hidup anda? Ya/Tidak (lingkari salah satu) | |
| ***Jika anda menjawab “ya”***, Agama apakah yang menginspirasi anda? (bisa menjawab lebih dari satu) | |
| - 1. Budha   2. Hindu   3. Islam | - 1. Kristen   2. Aliran kepercayaan lain: ______________ (tuliskan) |
| 1. A. Seberapa besar pemasukan per bulan anda? (lingkari salah satu di bawah ini) | |
| - 1. Dibawah standar gaji minimum di negara saya   2. Sebesar standar gaji minimum di negara saya   3. Sebesar standar gaji rata-rata di negara saya | - 1. Dua kali lipat standar gaji rata-rata di negara saya   2. Lebih dari dua kali lipat standar gaji rata-rata di negara saya   3. Tidak menjawab |
| B. Seberapa besar pengeluaran per bulan anda? | |
| - 1. Kurang dari Rp. 5.000.000,-   2. Antara Rp. 5.000.001,- sampai Rp. 10.000.000,-   3. Antara Rp.10.000.001,- sampai Rp. 15.000.000,- | - 1. Antara Rp. 15.000.001,- sampai Rp. 25.000.000,-   2. Diatas Rp. 25.000.001,-   3. Tidak ingin menjawab |
| 1. Seberapa sering anda memakan daging (termasuk ikan) dalam seminggu? (lingkari salah satu di bawah ini) | |
| - 1. Saya tidak makan daging   2. Sekali seminggu   3. 2-3 hari dalam seminggu | - 1. 4-6 hari dalam seminggu   2. Setiap hari |
| 1. Apakah anda mempunyai hewan peliharaan? **Ya/Tidak** (lingkari salah satu) | |
| ***Jika anda menjawab “ya”***, hewan peliharaan apa yang anda punya? (bisa menjawab lebih dari satu) | |
| - 1. Kucing   2. Anjing   3. Ikan   4. Burung   5. Reptil | - 1. Sejenis tikus (contoh: tikus putih, marmut, hamster, dll)   2. Ayam, Bebek/Angsa, dll   3. Kuda   4. Lain-lain: ________________ (tuliskan) |
| 1. Seberapa sering anda mengunjungi kebun binatang atau akuarium umum? (lingkari salah satu di bawah ini) | |
| - 1. Sekali sebulan   2. Sekali dalam enam bulan   3. Sekali setahun | - 1. Sekali dalam dua tahun atau lebih dari dua tahun   2. Tidak pernah |

## **Pemeriksaan perlakuan kelompok narasi**

| Pada bagian ini, hitamkan HANYA SATU lingkaran.  Anda akan menemukan dua pernyataan di setiap sisi yang berlawanan (kiri dan kanan). Hitamkan lingkaran mendekati pernyataan yang paling bisa anda terima. |
| --- |
| Contoh:   \| Hampir semua guru di sekolah saya baik \| **◌ ◌ ◌ ◌** \| Hampir semua guru di sekolah saya tegas \| \| --- \| --- \| --- \|   *Jika anda* ***mutlak merasa*** *kebanyakan guru di sekolah anda tegas daripada baik, anda dapat mengisi lingkaran yang paling dekat dengan pernyataan itu (lihat contoh dibawah)*   \| Hampir semua guru di sekolah saya baik \| **◌ ◌ ◌ •** \| Hampir semua guru di sekolah saya tegas \| \| --- \| --- \| --- \|   *Jika anda merasa kebanyakan guru* ***lebih cenderung*** *baik daripada tegas, anda dapat mengisi seperti dibawah ini:*   \| Hampir semua guru di sekolah saya baik \| **◌ • ◌ ◌** \| Hampir semua guru di sekolah saya tegas \| \| --- \| --- \| --- \|   *PERHATIAN: Anda hanya diperbolehkan mengisi satu lingkaran.*  Catatan: Semakin dekat posisi lingkaran yang anda isi ke pernyataan, semakin kuat anda merasa pernyataan itu menggambarkan anda. |

| 01. | Manusia ditinggikan derajatnya dari makhluk-makhluk yang lain | **◌ ◌ ◌ ◌** | Kerusakan lingkungan tinggi dan mengkhawatirkan |
| --- | --- | --- | --- |
| 02. | Manusia diutus untuk memimpin | **◌ ◌ ◌ ◌** | Sumber daya alam terancam habis |
| 03. | Manusia ditinggikan derajatnya dari makhluk-makhluk yang lain | **◌ ◌ ◌ ◌** | Tuhan mengutus manusia untuk menjaga |
| 04. | Manusia perlu menafkahi keluarganya | **◌ ◌ ◌ ◌** | Manusia perlu menjaga dan merawat alam dan lingkungan |
| 05. | Tuhan mengutus manusia untuk memimpin | **◌ ◌ ◌ ◌** | Kesejahteraan satwa (melindungi semua makhluk hidup) |
| 06. | Tuhan mengutus manusia untuk menjaga | **◌ ◌ ◌ ◌** | Manusia diutus untuk memimpin |
| 07. | Tuhan mengutus manusia untuk memimpin | **◌ ◌ ◌ ◌** | Binatang membutuhkan tempat hidup |
| 08. | Manusia kurang berserah dan terlalu khawatir | **◌ ◌ ◌ ◌** | Kesejahteraan satwa (melindungi semua makhluk hidup) |
| 09. | Tuhan menciptakan manusia sebagai makhluk mulia | **◌ ◌ ◌ ◌** | Tuhan mengutus manusia untuk menjaga |
| 10. | Tuhan mengutus manusia untuk memimpin | **◌ ◌ ◌ ◌** | Alam menghidupi manusia |

## **Ecocentric-Antropocentric-Apathy (Thompson & Barton, 1994)**

| Berikan angka 1 (satu) sampai dengan 5 (lima) untuk menunjukkan setuju atau tidaknya anda terhadap suatu pernyataan menurut jenjang di bawah ini:   \| 1 \| 2 \| 3 \| 4 \| 5 \| \| --- \| --- \| --- \| --- \| --- \| \| Sangat tidak setuju \| Tidak setuju \| Netral \| Setuju \| Sangat setuju \| |
| --- | --- | --- | --- | --- | --- | --- | --- | --- | --- | --- |
| Contoh:  Saya tidak pernah lupa mengunci pintu __________  *Jika anda ‘setuju’ dengan pertanyaan diatas maka anda mengisinya dengan angka ‘4’*  Saya tidak pernah lupa mengunci pintu _____4____  Catatan: Semakin tinggi angka yang anda bubuhkan berarti semakin setuju anda dengan pernyataan itu. |

| 1. Salah satu hal terburuk tentang kelebihan populasi adalah banyak daerah alami yang hancur untuk pembangunan | ____ |
| --- | --- |
| 1. Tanpa alasan lain selain hanya untuk berada di alam, saya senang pergi menikmati waktu di alam liar. | ____ |
| 1. Ancaman lingkungan seperti penggundulan hutan dan menipisnya ozon itu cenderung dibesar-besarkan | ____ |
| 1. A. Hal terburuk akibat hilangnya hutan adalah membatasi pengembangan obat baru | ____ |
| 1. B. Hal terburuk akibat hilangnya hutan adalah mengurangi tanaman dan satwa yang berguna untuk manusia. | ____ |
| 1. Terkadang saya sedih melihat hutan dibuka untuk pertanian | ____ |
| 1. Menurut saya, kebanyakan orang-orang pelestarian alam adalah orang yang pesimis dan terlalu takut. | ____ |
| 1. Saya lebih memilih daerah alam suaka margasatwa daripada kebun binatang | ____ |
| 1. Saya pikir masalah menipisnya sumber daya alam tidak seburuk yang dikemukakan banyak orang. | ____ |
| 1. Sulit bagi saya menemukan alasan untuk terlalu khawatir dengan isu lingkungan. | ____ |
| 1. Saya terganggu dengan keadaan manusia kehabisan pasokan minyak. | ____ |
| 1. Bagi saya, untuk bahagia, salah satunya adalah kebutuhan untuk pergi menikmati waktu di alam | ____ |
| 1. Hal yang paling mengkhawatirkan saya soal kegundulan hutan adalah tidak akan tersedia cukup kayu untuk generasi mendatang | ____ |
| 1. Saya tidak merasa bahwa manusia bergantung pada alam untuk bertahan hidup | ____ |
| 1. Terkadang saat saya tidak bahagia, saya menemukan kenyamanan di alam | ____ |
| 1. Seiring waktu, sebagian besar masalah lingkungan akan terselesaikan dengan sendirinya. | ____ |
| 1. Saya tidak peduli masalah lingkungan. | ____ |
| 1. Saya menentang program untuk melestarikan alam liar, mengurangi polusi dan melestarikan sumber daya | ____ |
| 1. Menyaksikan kerusakan lingkungan alam membuat saya sedih | ____ |
| 1. Kelangsungan hidup manusia adalah alasan paling penting perlunya pelestarian alam | ____ |
| 1. Salah satu hal terbaik tentang daur ulang adalah menghemat uang | ____ |
| 1. Alam itu penting karena sumbangannya pada kesenangan dan kesejahteraan manusia | ____ |
| 1. Terlalu banyak perhatian diberikan pada pelestrarian lingkungan | ____ |
| 1. Alam berharga untuk keberlangsungannya sendiri | ____ |
| 1. Kita perlu melestarikan sumber daya untuk mempertahankan kualitas hidup yang tinggi | ____ |
| 1. Bagi saya, berada di alam adalah penghilang stres yang hebat. | ____ |
| 1. Salah satu alasan terpenting untuk melestarikan adalah memastikan kesinambungan standar hidup yang tinggi | ____ |
| 1. Salah satu alasan paling penting untuk melestarikan adalah memelihara daerah liar | ____ |
| 1. Melanjutkan pengembangan lahan adalah ide bagus asalkan kualitas hidup yang tinggi bisa terjaga | ____ |
| 1. Terkadang hewan seperti manusia bagi saya. | ____ |
| 1. Sama seperti hewan, manusia adalah bagian dari ekosistemnya. | ____ |

## **The Animal Issue Scale (AIS)**

Berikan angka 1 (satu) sampai dengan 5 (lima) untuk menunjukkan dapat atau tidaknya anda menerima suatu pernyataan menurut jenjang dibawah ini:

| 1 | 2 | 3 | 4 | 5 |
| --- | --- | --- | --- | --- |
| Sangat tidak bisa diterima | Tidak bisa diterima | Ragu-ragu | Bisa diterima | Sangat bisa diterima |

**Catatan**: Semakin tinggi angka yang anda bubuhkan berarti semakin anda dapat menerima pernyataan itu.

| 1 Memelihara hewan untuk produksi makanan atau pakaian | ____ |
| --- | --- |
| 2 Hewan sebagai hewan peliharaan | ____ |
| 3 Memelihara hewan untuk pendidikan masyarakat di kebun binatang, taman margasatwa, dll. | ____ |
| 4 Menggunakan hewan untuk bekerja | ____ |
| 5 Menggunakan hewan untuk hiburan atau olahraga | ____ |
| 6 Melakukan operasi pada hewan untuk memperbaiki kesehatan hewan itu. | ____ |
| 7 Dekorasi hewan, seperti mewarnai atau memotong rambut mereka untuk alasan estetika | ____ |
| 8 Mengurangi seksualitas hewan dengan implan hormon | ____ |
| 9 Menghilangkan bagian tubuh, seperti pemotongan ekor atau kuku | ____ |
| 10 Menandai hewan dengan merk atau tindik kuping | ____ |
| 11 Menghilangkan jaringan mati, seperti menghilangkan rambut/wol atau pemangkasan kaki | ____ |
| 12 Membunuh hewan muda yang bergantung pada orang tua mereka | ____ |
| 13 Memungkinkan hewan mengalami rasa sakit saat disembelih | ____ |
| 14 Menggunakan mayat hewan sebagai produk setelah kematian alami mereka | ____ |
| 15 Membunuh hewan saat mereka terluka parah atau sakit | ____ |
| 16 Membunuh hewan peliharaan yang sehat dan tidak diinginkan karena kelebihan populasi | ____ |
| 17 Merampas hewan dari kebutuhan mereka akan makanan dan air | ____ |
| 18 Merampas hewan dari lingkungan yang layak untuk bernaung, termasuk tempat berlindung | ____ |
| 19 Menimbulkan rasa sakit, luka atau penyakit pada hewan | ____ |
| 20 Tidak menyediakan ruang yang cukup, fasilitas yang memadai dan kebersamaan dengan hewan lain yang dibutuhkan hewan | ____ |
| 21 Mengijinkan pada kondisi dan perawatan yang menyebabkan penderitaan mental | ____ |
| 22 Mengamati perilaku hewan dalam percobaan | ____ |
| 23 Percobaan untuk meningkatkan kesejahteraan atau kesehatan hewan | ____ |
| 24 Percobaan medis menggunakan hewan untuk memperbaiki kesehatan manusia | ____ |
| 25 Menguji kosmetik atau produk rumah tangga pada binatang | ____ |
| 26 Mengoperasikan hewan hidup untuk manfaat penelitian obat manusia | ____ |
| 27 Meningkatkan kemampuan produktif dan reproduksi hewan melalui perubahan genetik, misalnya sapi yang menghasilkan lebih banyak susu | ____ |
| 28 Meningkatkan ketahanan penyakit atau kesehatan hewan melalui pengubahan genetiknya | ____ |
| 29 Menciptakan hewan ternak yang lebih menguntungkan karena menganggap mereka (hewan) merasa senang dengan sedikitnya kegiatan dan keinginan untuk aktif | ____ |
| 30 Pemilihan genetik hewan peliharaan, seperti anjing dan kucing, untuk meningkatkan kelangkaannya atau meningkatkan nilai silsilah asal-usulnya. | ____ |
| 31 Modifikasi genetik tanaman untuk makanan hewani | ____ |
| 32 Membunuh hewan karena mereka bukan hewan asli di linkungan tinggal mereka | ____ |
| 33 Membunuh hewan liar untuk menghentikan penyebaran penyakit yang bisa menyerang manusia | ____ |
| 34 Mengendalikan populasi satwa liar dengan membunuh | ____ |
| 35 Mengontrol populasi hewan melalui sterilisasi | ____ |
| 36 Menghancurkan linkungan habitat spesies hewan yang terancam punah | ____ |
| 37 Menghancurkan lingkungan habitat spesies hewan yang tidak terancam punah guna mengembangkan dan mempromosikan urbanisasi atau tanaman pangan untuk memberi makan manusia | ____ |
| 38 Pengorbanan binatang dalam upacara keagamaan | ____ |
| 39 Mempertimbangkan beberapa spesies hewan sebagai simbol kesucian atau keberuntungan. | ____ |
| 40 Mempertimbangkan beberapa spesies hewan sebagai jahat atau nasib buruk | ____ |
| 41 Orangtua menampilkan perlakuan kejam terhadap hewan di depan anak-anak mereka | ____ |
| 42 Menimbulkan rasa sakit atau luka pada hewan sebagai bagian dari tradisi budaya | ____ |
| 43 Menduplikasi hewan untuk keuntungan manusia | ____ |

## **The Religious Orientation Scale (ROS)**

Berikan angka 1 (satu) sampai dengan 5 (lima) untuk menunjukkan setuju atau tidaknya anda terhadap suatu pernyataan menurut jenjang di bawah ini:

| 1 | 2 | 3 | 4 | 5 |
| --- | --- | --- | --- | --- |
| Sangat tidak setuju | Tidak setuju | Ragu-ragu | Setuju | Sangat setuju |

**Catatan:** Semakin tinggi angka yang anda bubuhkan berarti semakin setuju anda dengan pernyataan itu.

| 1. Saya berusaha keras untuk menjalani hidup saya sesuai dengan keyakinan agamawi saya | ____ |
| --- | --- |
| 1. Tidak masalah apa yang saya percayai selama saya baik | ____ |
| 1. Saya sering mengalami perasaan kuat adanya kehadiran ilahi | ____ |
| 1. Seluruh pendekatan saya pada hidup didasarkan pada agama saya | ____ |
| 1. Do’a yang saya panjatkan ketika sendirian sama pentingnya dengan yang saya panjatkan ketika beribadah berjamaah di rumah ibadah (contoh: masjid, gereja, pura, wihara, dll). | ____ |
| 1. Dalam seminggu, saya datang ke rumah ibadah sekali atau bisa lebih | ____ |
| 1. Agama saya penting karena menjawab banyak pertanyaan mengenai makna kehidupan | ____ |
| 1. Saya senang membaca bacaan mengenai agama saya. | ____ |
| 1. Sangat penting bagi saya untuk menghabiskan waktu dalam pemikiran dan Do’a pribadi. | ____ |
| 1. Bagi saya, agama terutama memberikan kenyamanan ketika ada masalah dan ketika mengalami kesedihan. | ____ |
| 1. Doa adalah untuk kedamaian dan kebahagiaan. | ____ |
| 1. Saya berdoa terutama untuk mendapatkan kelegaan dan perlindungan | ____ |
| 1. Saya datang ke rumah ibadah karena membantu saya mendapat teman. | ____ |
| 1. Saya datang ke rumah ibadah terutama karena saya senang menemui orang-orang yang saya kenal disana | ____ |
| 1. Saya datang ke rumah ibadah seringkali untuk menghabiskan waktu bersama teman-teman saya. | ____ |

## **The Ethical Position Questionnaire (ROS)**

Berikan angka 1 (satu) sampai dengan 9 (sembilan) untuk menunjukkan setuju atau tidaknya anda terhadap suatu pernyataan menurut jenjang di bawah ini:

| 1 | 2 | 3 | 4 | 5 | 6 | 7 | 8 | 9 |
| --- | --- | --- | --- | --- | --- | --- | --- | --- |
| Sepenuhnya tidak setuju | Tidak setuju | Agak tidak setuju | Sedikit tidak setuju | Ragu-ragu | Sedikit setuju | Agak setuju | Setuju | Sepenuhnya setuju |

**Catatan**: Semakin tinggi angka yang anda bubuhkan berarti semakin setuju anda dengan pernyataan itu.

| 1. Orang-orang baiknya dapat memastikan bahwa tindakan mereka tidak pernah ditujukan untuk menyakiti orang lain, sekecil apapun itu. | ____ |
| --- | --- |
| 1. Sekecil apapun, membawa resiko kepada orang lain sebaiknya tidak boleh ditolerir. | ____ |
| 1. Apapun manfaat yang bisa didapat, adanya kemungkinan menyakiti orang lain itu selalu salah. | ____ |
| 1. Baik secara psikologis maupun fisik, seseorang seharusnya tidak menyakiti orang lain. | ____ |
| 1. Seseorang seharusnya tidak melakukan tindakan apapun yang berkemungkinan mengancam martabat dan kesejahteraan orang lain. | ____ |
| 1. Jika bisa merugikan orang yang tidak bersalah, maka sebaiknya tindakan itu tidak dilakukan. | ____ |
| 1. Memutuskan melakukan tindakan dengan pertimbangan menyeimbangkan akibat positif dan negatif dari tindakan adalah tidak bermoral. | ____ |
| 1. Martabat dan kesejahteraan orang banyak harus menjadi perhatian yang paling penting di masyarakat manapun. | ____ |
| 1. Mengorbankan kesejahteraan orang lain tidak pernah diperlukan | ____ |
| 1. Perilaku moral adalah berbagai tindakan yang sangat sesuai dengan cita-cita dari hampir semua tindakan "sempurna". | ____ |
| 1. Tiada prinsip etika sebegitu pentingnya sehingga harus dijadikan komponen kode etik apapun. | ____ |
| 1. Apa yang etis dan tidak di suatu situasi dan masyarakat bisa berbeda di situasi dan masyarakat lain. | ____ |
| 1. Standar moral harus dilihat sebagai sesuatu yang khas; Apa yang seseorang anggap sebagai bermoral boleh jadi dianggap tidak bermoral oleh orang lain. | ____ |
| 1. Jenis moralitas yang berbeda tidak dapat diperbandingkan kebenarannya. | ____ |
| 1. Apa yang etis untuk semua orang tidak akan pernah bisa terjawab karena pertimbangan apa yang bermoral dari tidak bermoral itu terserah kepada perseorangan. | ____ |
| 1. Standar moral hanyalah peraturan pribadi yang menunjukkan bagaimana seseorang harus bersikap, dan tidak dapat diterapkan dalam membuat penilaian terhadap orang lain. | ____ |
| 1. Pertimbangan etis dalam hubungan antar pribadi sangat rumit sehingga individu harus diijinkan untuk merumuskan kode mereka sendiri. | ____ |
| 1. Menerjemahkan posisi etis secara kaku sehingga mencegah beberapa jenis tindakan dapat menghalangi hubungan dan penyesuaian antar sesama yang lebih baik. | ____ |
| 1. Tidak ada aturan tentang kebohongan yang bisa dirumuskan; apakah kebohongan diperbolehkan atau tidak itu benar-benar tergantung pada situasi. | ____ |
| 1. Apakah kebohongan dinilai sebagai moral atau tidak bermoral tergantung pada situasi dan kondisi yang mendasari tindakan tersebut. | ____ |
