# Supplementary material for: The role of religious narratives and religious orientation towards concerns for the natural environment and animal welfare
Source: PLoS One. 2022 Aug 11;17(8):e0271515. doi: 10.1371/journal.pone.0271515 (PMC9371258; doi:10.1371/journal.pone.0271515)
Supplement: S5 File — (DOCX) [file pone.0271515.s005.docx]

Table 1 Manova between-subject factors

| **Between-Subjects Factors** | | | |
| --- | --- | --- | --- |
|  | | Value Label | N |
| Priming Narration | 1 | Stewardship (1) | 148 |
|  | 2 | Human domination (2) | 188 |
|  | 3 | No Narration (3) | 321 |

Table 2 Manova Multivatiate tests

| **Multivariate Tests^a^** | | | | | | |
| --- | --- | --- | --- | --- | --- | --- |
| Effect | | Value | F | Hypothesis df | Error df | Sig. |
| Intercept | Pillai's Trace | .992 | 7558.293^b^ | 10.000 | 645.000 | .000 |
|  | Wilks' Lambda | .008 | 7558.293^b^ | 10.000 | 645.000 | .000 |
|  | Hotelling's Trace | 117.183 | 7558.293^b^ | 10.000 | 645.000 | .000 |
|  | Roy's Largest Root | 117.183 | 7558.293^b^ | 10.000 | 645.000 | .000 |
| narasi | Pillai's Trace | .050 | 1.661 | 20.000 | 1292.000 | .034 |
|  | Wilks' Lambda | .950 | 1.661^b^ | 20.000 | 1290.000 | .033 |
|  | Hotelling's Trace | .052 | 1.662 | 20.000 | 1288.000 | .033 |
|  | Roy's Largest Root | .036 | 2.344^c^ | 10.000 | 646.000 | .010 |
| a. Design: Intercept + narasi | | | | | | |
| b. Exact statistic | | | | | | |
| c. The statistic is an upper bound on F that yields a lower bound on the significance level. | | | | | | |

Table 3 Tests of Between-Subjects Effects

| **Tests of Between-Subjects Effects** | | | | | | |
| --- | --- | --- | --- | --- | --- | --- |
| Source | Dependent Variable | Type III Sum of Squares | df | Mean Square | F | Sig. |
| Corrected Model | Apathy_ALL_INV_ORI | 5.883^a^ | 2 | 2.942 | 5.707 | .003 |
|  | AIS_ALL_ORI | 3.417^b^ | 2 | 1.708 | 6.132 | .002 |
|  | AIS_AnimUse_ORI | .853^c^ | 2 | .426 | .989 | .372 |
|  | AIS_Intgrty_ORI | 6.808^d^ | 2 | 3.404 | 5.411 | .005 |
|  | AIS_KillWelfare_ORI | 3.724^e^ | 2 | 1.862 | 3.048 | .048 |
|  | AIS_Xprmnt_ORI | 2.987^f^ | 2 | 1.493 | 2.093 | .124 |
|  | AIS_Genchng_ORI | 2.714^g^ | 2 | 1.357 | 2.481 | .084 |
|  | AIS_EnvIss_ORI | 6.252^h^ | 2 | 3.126 | 4.890 | .008 |
|  | AIS_SocAtt_ORI | 3.267^i^ | 2 | 1.633 | 2.735 | .066 |
|  | ROS_PersIntExt_ORI | 1.218^j^ | 2 | .609 | 2.145 | .118 |
|  | ROS_Soc_ORI | 2.067^k^ | 2 | 1.034 | 1.017 | .362 |
| Intercept | Apathy_ALL_INV_ORI | 3763.463 | 1 | 3763.463 | 7301.131 | .000 |
|  | AIS_ALL_ORI | 3872.229 | 1 | 3872.229 | 13897.201 | .000 |
|  | AIS_AnimUse_ORI | 5811.092 | 1 | 5811.092 | 13483.053 | .000 |
|  | AIS_Intgrty_ORI | 3425.799 | 1 | 3425.799 | 5445.827 | .000 |
|  | AIS_KillWelfare_ORI | 2619.919 | 1 | 2619.919 | 4288.575 | .000 |
|  | AIS_Xprmnt_ORI | 5344.830 | 1 | 5344.830 | 7492.529 | .000 |
|  | AIS_Genchng_ORI | 6427.790 | 1 | 6427.790 | 11753.312 | .000 |
|  | AIS_EnvIss_ORI | 3358.168 | 1 | 3358.168 | 5254.100 | .000 |
|  | AIS_SocAtt_ORI | 2701.650 | 1 | 2701.650 | 4523.234 | .000 |
|  | ROS_PersIntExt_ORI | 10627.027 | 1 | 10627.027 | 37421.496 | .000 |
|  | ROS_Soc_ORI | 4749.476 | 1 | 4749.476 | 4674.489 | .000 |
| narasi | Apathy_ALL_INV_ORI | 5.883 | 2 | 2.942 | 5.707 | .003 |
|  | AIS_ALL_ORI | 3.417 | 2 | 1.708 | 6.132 | .002 |
|  | AIS_AnimUse_ORI | .853 | 2 | .426 | .989 | .372 |
|  | AIS_Intgrty_ORI | 6.808 | 2 | 3.404 | 5.411 | .005 |
|  | AIS_KillWelfare_ORI | 3.724 | 2 | 1.862 | 3.048 | .048 |
|  | AIS_Xprmnt_ORI | 2.987 | 2 | 1.493 | 2.093 | .124 |
|  | AIS_Genchng_ORI | 2.714 | 2 | 1.357 | 2.481 | .084 |
|  | AIS_EnvIss_ORI | 6.252 | 2 | 3.126 | 4.890 | .008 |
|  | AIS_SocAtt_ORI | 3.267 | 2 | 1.633 | 2.735 | .066 |
|  | ROS_PersIntExt_ORI | 1.218 | 2 | .609 | 2.145 | .118 |
|  | ROS_Soc_ORI | 2.067 | 2 | 1.034 | 1.017 | .362 |
| Error | Apathy_ALL_INV_ORI | 337.113 | 654 | .515 |  |  |
|  | AIS_ALL_ORI | 182.226 | 654 | .279 |  |  |
|  | AIS_AnimUse_ORI | 281.869 | 654 | .431 |  |  |
|  | AIS_Intgrty_ORI | 411.411 | 654 | .629 |  |  |
|  | AIS_KillWelfare_ORI | 399.533 | 654 | .611 |  |  |
|  | AIS_Xprmnt_ORI | 466.534 | 654 | .713 |  |  |
|  | AIS_Genchng_ORI | 357.667 | 654 | .547 |  |  |
|  | AIS_EnvIss_ORI | 418.005 | 654 | .639 |  |  |
|  | AIS_SocAtt_ORI | 390.623 | 654 | .597 |  |  |
|  | ROS_PersIntExt_ORI | 185.724 | 654 | .284 |  |  |
|  | ROS_Soc_ORI | 664.491 | 654 | 1.016 |  |  |
| Total | Apathy_ALL_INV_ORI | 4610.222 | 657 |  |  |  |
|  | AIS_ALL_ORI | 4520.927 | 657 |  |  |  |
|  | AIS_AnimUse_ORI | 6732.375 | 657 |  |  |  |
|  | AIS_Intgrty_ORI | 4286.641 | 657 |  |  |  |
|  | AIS_KillWelfare_ORI | 3354.534 | 657 |  |  |  |
|  | AIS_Xprmnt_ORI | 6412.078 | 657 |  |  |  |
|  | AIS_Genchng_ORI | 7526.996 | 657 |  |  |  |
|  | AIS_EnvIss_ORI | 4193.292 | 657 |  |  |  |
|  | AIS_SocAtt_ORI | 3426.960 | 657 |  |  |  |
|  | ROS_PersIntExt_ORI | 11930.176 | 657 |  |  |  |
|  | ROS_Soc_ORI | 5898.018 | 657 |  |  |  |
| Corrected Total | Apathy_ALL_INV_ORI | 342.996 | 656 |  |  |  |
|  | AIS_ALL_ORI | 185.643 | 656 |  |  |  |
|  | AIS_AnimUse_ORI | 282.722 | 656 |  |  |  |
|  | AIS_Intgrty_ORI | 418.219 | 656 |  |  |  |
|  | AIS_KillWelfare_ORI | 403.257 | 656 |  |  |  |
|  | AIS_Xprmnt_ORI | 469.521 | 656 |  |  |  |
|  | AIS_Genchng_ORI | 360.381 | 656 |  |  |  |
|  | AIS_EnvIss_ORI | 424.257 | 656 |  |  |  |
|  | AIS_SocAtt_ORI | 393.890 | 656 |  |  |  |
|  | ROS_PersIntExt_ORI | 186.942 | 656 |  |  |  |
|  | ROS_Soc_ORI | 666.558 | 656 |  |  |  |
| a. R Squared = .017 (Adjusted R Squared = .014) | | | | | | |
| b. R Squared = .018 (Adjusted R Squared = .015) | | | | | | |
| c. R Squared = .003 (Adjusted R Squared = .000) | | | | | | |
| d. R Squared = .016 (Adjusted R Squared = .013) | | | | | | |
| e. R Squared = .009 (Adjusted R Squared = .006) | | | | | | |
| f. R Squared = .006 (Adjusted R Squared = .003) | | | | | | |
| g. R Squared = .008 (Adjusted R Squared = .004) | | | | | | |
| h. R Squared = .015 (Adjusted R Squared = .012) | | | | | | |
| i. R Squared = .008 (Adjusted R Squared = .005) | | | | | | |
| j. R Squared = .007 (Adjusted R Squared = .003) | | | | | | |
| k. R Squared = .003 (Adjusted R Squared = .000) | | | | | | |

Table 4 Bonferroni multiple comparison

| **Multiple Comparisons** | | | | | | | |
| --- | --- | --- | --- | --- | --- | --- | --- |
| Dependent Variable | (I) Priming Narration | (J) Priming Narration | Mean Difference (I-J) | Std. Error | Sig. | 95% Confidence Interval | |
|  |  |  |  |  |  | Lower Bound | Upper Bound |
| Apathy_ALL_INV_ORI | Stewardship (1) | Human domination (2) | -.1902^*^ | .07890 | .049 | -.3795 | -.0008 |
|  |  | No Narration (3) | -.2391^*^ | .07133 | .003 | -.4103 | -.0678 |
|  | Human domination (2) | Stewardship (1) | .1902^*^ | .07890 | .049 | .0008 | .3795 |
|  |  | No Narration (3) | -.0489 | .06594 | 1.000 | -.2071 | .1094 |
|  | No Narration (3) | Stewardship (1) | .2391^*^ | .07133 | .003 | .0678 | .4103 |
|  |  | Human domination (2) | .0489 | .06594 | 1.000 | -.1094 | .2071 |
| AIS_ALL_ORI | Stewardship (1) | Human domination (2) | -.1993^*^ | .05801 | .002 | -.3386 | -.0601 |
|  |  | No Narration (3) | -.1393^*^ | .05245 | .024 | -.2652 | -.0134 |
|  | Human domination (2) | Stewardship (1) | .1993^*^ | .05801 | .002 | .0601 | .3386 |
|  |  | No Narration (3) | .0600 | .04848 | .648 | -.0563 | .1764 |
|  | No Narration (3) | Stewardship (1) | .1393^*^ | .05245 | .024 | .0134 | .2652 |
|  |  | Human domination (2) | -.0600 | .04848 | .648 | -.1764 | .0563 |
| AIS_AnimUse_ORI | Stewardship (1) | Human domination (2) | -.0987 | .07214 | .515 | -.2718 | .0745 |
|  |  | No Narration (3) | -.0385 | .06523 | 1.000 | -.1950 | .1181 |
|  | Human domination (2) | Stewardship (1) | .0987 | .07214 | .515 | -.0745 | .2718 |
|  |  | No Narration (3) | .0602 | .06029 | .955 | -.0845 | .2049 |
|  | No Narration (3) | Stewardship (1) | .0385 | .06523 | 1.000 | -.1181 | .1950 |
|  |  | Human domination (2) | -.0602 | .06029 | .955 | -.2049 | .0845 |
| AIS_Intgrty_ORI | Stewardship (1) | Human domination (2) | -.2681^*^ | .08716 | .007 | -.4773 | -.0589 |
|  |  | No Narration (3) | -.2222^*^ | .07880 | .015 | -.4114 | -.0331 |
|  | Human domination (2) | Stewardship (1) | .2681^*^ | .08716 | .007 | .0589 | .4773 |
|  |  | No Narration (3) | .0458 | .07284 | 1.000 | -.1290 | .2207 |
|  | No Narration (3) | Stewardship (1) | .2222^*^ | .07880 | .015 | .0331 | .4114 |
|  |  | Human domination (2) | -.0458 | .07284 | 1.000 | -.2207 | .1290 |
| AIS_KillWelfare_ORI | Stewardship (1) | Human domination (2) | -.1967 | .08589 | .067 | -.4028 | .0095 |
|  |  | No Narration (3) | -.1664 | .07766 | .098 | -.3528 | .0200 |
|  | Human domination (2) | Stewardship (1) | .1967 | .08589 | .067 | -.0095 | .4028 |
|  |  | No Narration (3) | .0303 | .07178 | 1.000 | -.1420 | .2026 |
|  | No Narration (3) | Stewardship (1) | .1664 | .07766 | .098 | -.0200 | .3528 |
|  |  | Human domination (2) | -.0303 | .07178 | 1.000 | -.2026 | .1420 |
| AIS_Xprmnt_ORI | Stewardship (1) | Human domination (2) | -.1872 | .09281 | .132 | -.4100 | .0356 |
|  |  | No Narration (3) | -.0820 | .08392 | .987 | -.2834 | .1194 |
|  | Human domination (2) | Stewardship (1) | .1872 | .09281 | .132 | -.0356 | .4100 |
|  |  | No Narration (3) | .1052 | .07757 | .526 | -.0810 | .2914 |
|  | No Narration (3) | Stewardship (1) | .0820 | .08392 | .987 | -.1194 | .2834 |
|  |  | Human domination (2) | -.1052 | .07757 | .526 | -.2914 | .0810 |
| AIS_Genchng_ORI | Stewardship (1) | Human domination (2) | -.1804 | .08127 | .080 | -.3754 | .0147 |
|  |  | No Narration (3) | -.1120 | .07348 | .384 | -.2884 | .0643 |
|  | Human domination (2) | Stewardship (1) | .1804 | .08127 | .080 | -.0147 | .3754 |
|  |  | No Narration (3) | .0684 | .06792 | .944 | -.0947 | .2314 |
|  | No Narration (3) | Stewardship (1) | .1120 | .07348 | .384 | -.0643 | .2884 |
|  |  | Human domination (2) | -.0684 | .06792 | .944 | -.2314 | .0947 |
| AIS_EnvIss_ORI | Stewardship (1) | Human domination (2) | -.2729^*^ | .08785 | .006 | -.4838 | -.0621 |
|  |  | No Narration (3) | -.1753 | .07943 | .083 | -.3659 | .0154 |
|  | Human domination (2) | Stewardship (1) | .2729^*^ | .08785 | .006 | .0621 | .4838 |
|  |  | No Narration (3) | .0976 | .07342 | .552 | -.0786 | .2739 |
|  | No Narration (3) | Stewardship (1) | .1753 | .07943 | .083 | -.0154 | .3659 |
|  |  | Human domination (2) | -.0976 | .07342 | .552 | -.2739 | .0786 |
| AIS_SocAtt_ORI | Stewardship (1) | Human domination (2) | -.1921 | .08493 | .072 | -.3960 | .0117 |
|  |  | No Narration (3) | -.1432 | .07679 | .188 | -.3275 | .0411 |
|  | Human domination (2) | Stewardship (1) | .1921 | .08493 | .072 | -.0117 | .3960 |
|  |  | No Narration (3) | .0489 | .07098 | 1.000 | -.1214 | .2193 |
|  | No Narration (3) | Stewardship (1) | .1432 | .07679 | .188 | -.0411 | .3275 |
|  |  | Human domination (2) | -.0489 | .07098 | 1.000 | -.2193 | .1214 |
| ROS_PersIntExt_ORI | Stewardship (1) | Human domination (2) | -.0753 | .05856 | .596 | -.2159 | .0652 |
|  |  | No Narration (3) | .0254 | .05295 | 1.000 | -.1017 | .1525 |
|  | Human domination (2) | Stewardship (1) | .0753 | .05856 | .596 | -.0652 | .2159 |
|  |  | No Narration (3) | .1007 | .04894 | .120 | -.0168 | .2182 |
|  | No Narration (3) | Stewardship (1) | -.0254 | .05295 | 1.000 | -.1525 | .1017 |
|  |  | Human domination (2) | -.1007 | .04894 | .120 | -.2182 | .0168 |
| ROS_Soc_ORI | Stewardship (1) | Human domination (2) | -.0997 | .11077 | 1.000 | -.3656 | .1662 |
|  |  | No Narration (3) | .0313 | .10015 | 1.000 | -.2091 | .2716 |
|  | Human domination (2) | Stewardship (1) | .0997 | .11077 | 1.000 | -.1662 | .3656 |
|  |  | No Narration (3) | .1310 | .09257 | .473 | -.0912 | .3532 |
|  | No Narration (3) | Stewardship (1) | -.0313 | .10015 | 1.000 | -.2716 | .2091 |
|  |  | Human domination (2) | -.1310 | .09257 | .473 | -.3532 | .0912 |
| Based on observed means.  The error term is Mean Square(Error) = 1.016. | | | | | | | |
| *. The mean difference is significant at the .05 level. | | | | | | | |
