# Supplementary material for: The role of religious narratives and religious orientation towards concerns for the natural environment and animal welfare
Source: PLoS One. 2022 Aug 11;17(8):e0271515. doi: 10.1371/journal.pone.0271515 (PMC9371258; doi:10.1371/journal.pone.0271515)
Supplement: S6 File — (DOCX) [file pone.0271515.s006.docx]

Table 1 Main variables with demographic and other important determinants to GEA

|  | | GEA | | | | Effect size | | | CI (95%) | |
| --- | --- | --- | --- | --- | --- | --- | --- | --- | --- | --- |
|  |  | b | Std. b | | |  | | | Lower | Upper |
| Model 1^A^ : Main variables | |  |  |  |  | |  |  | |  |
| R = 0.386 | (Constant) | 3.55 |  | ** |  | |  | 3.12 | | 3.98 |
| R2 = 0.149 | Stewardship priming narrative group^1^? Yes (1) – No (0) | -0.24 | -0.14 | ** | 0.33^D^ | | + | -0.37 | | -0.11 |
| df =4, 652 | Human Dominance priming narrative group^1^? Yes (1) – No (0) | -0.04 | -0.02 |  | 0.00^C^ | |  | -0.16 | | 0.08 |
|  | ROS Personal | -0.35 | -0.26 | ** | 0.07^C^ | | + | -0.44 | | -0.25 |
|  | ROS Social | 0.19 | 0.27 | ** | 0.08^C^ | | + | 0.14 | | 0.24 |
| Model 2^B^: Main variables with demographic and other determinants | | | |  |  | |  |  | |  |
| R = 0.465 | (Constant) | 3.14 |  | ** |  | |  | 2.33 | | 3.95 |
| R2 = 0.216 | ROS Personal | -0.29 | -0.21 | ** | 0.04 | | + | -0.41 | | -0.16 |
| df =39, 402 | ROS Social | 0.14 | 0.21 | ** | 0.04 | | + | 0.08 | | 0.21 |
|  | How often do you consume meat in a week^2^? I don't consume meat: Yes (1) – No (0) | 0.39 | 0.14 | ** | 0.02 | |  | 0.12 | | 0.66 |
|  | Stewardship priming narrative group^1^? Yes (1) – No (0) | -0.17 | -0.10 | * | 0.33^D^ | | + | -0.34 | | -0.00 |
|  | What is the highest level of schooling you have completed? Senior high: Yes (1) – No (0) | -0.29 | -0.11 |  | 0.01^C^ | |  | -0.58 | | 0.01 |
|  | What is your marriage status? Married: Yes (1) – No (0) | -0.25 | -0.14 |  | 0.01^C^ | |  | -0.50 | | 0.01 |
|  | How often do you visit a zoo or aquarium? Once every six month: Yes (1) – No (0) | -0.25 | -0.10 |  | 0.01^C^ | |  | -0.52 | | 0.02 |
|  | Do you have any affiliation to religious organization? Yes (1) – No (0) | 0.13 | 0.09 |  | 0.01^C^ | |  | -0.01 | | 0.27 |
|  | How often do you consume meat in a week? Four to six days a week: Yes (1) – No (0) | -0.18 | -0.09 |  | 0.01^C^ | |  | -0.39 | | 0.03 |
|  | What is your gross household expenses per month? Refuse to answer: Yes (1) – No (0) | 0.15 | 0.09 |  | 0.01^C^ | |  | -0.03 | | 0.33 |
|  | What is your gross household income per month? About the average income in my country: Yes (1) – No (0) | -0.13 | -0.07 |  | 0.00^C^ | |  | -0.32 | | 0.07 |
|  | In what sort of house do you live? Own house: Yes (1) – No (0) | 0.09 | 0.07 |  | 0.00^C^ | |  | -0.07 | | 0.25 |
|  | How often do you visit a zoo or aquarium? Once every two or more year: Yes (1) – No (0) | -0.10 | -0.07 |  | 0.00^C^ | |  | -0.27 | | 0.08 |
|  | How often do you consume meat in a week? Everyday: Yes (1) – No (0) | -0.11 | -0.05 |  | 0.00^C^ | |  | -0.33 | | 0.11 |
|  | What is your gross household expenses per month? Above 25 million: Yes (1) – No (0) | -0.46 | -0.04 |  | 0.00^C^ | |  | -1.39 | | 0.47 |
|  | In what sort of house do you live? Apartment: Yes (1) – No (0) | 0.29 | 0.05 |  | 0.00^C^ | |  | -0.29 | | 0.86 |

*p<.05; **p<.01; ^A^regression using enter method in a stepwise manner; ^B^regression using enter method; ^C^effect-size calculation using eta squared (F2); ^D^effect-size calculation using Hedge’s g; +small effect size F2>=0.02 (or in some cases of categorical dummy variable, using Cohen’s D/Hedges’g >= 0.2); ++medium effect size F2>=0.15 (or in some cases of categorical dummy variable, using cohen’s D/Hedges’g >=0.5); ^1^compared to respondents who fill the survey without having to read any narrative; ^2^compared to respondent who eat meat once a week.

Table 1 Main variables with demographic and other important determinants to GEA (continued)

|  | | GEA | | | | | Effect size | | | | | CI (95%) | | |
| --- | --- | --- | --- | --- | --- | --- | --- | --- | --- | --- | --- | --- | --- | --- |
|  |  | b | Std. b | | | |  | | | | | Lower | | Upper |
| Model 2^B^: Main variables with demographic and other determinants (continued) | | | | |  | | |  | |  | | |  |  |
|  | What is your gross household income per month? About the minimum income in my country: Yes (1) – No (0) | 0.09 | 0.05 |  | | 0.00^C^ | | |  | | -0.10 | | | 0.29 |
|  | Is religion important for you? Yes (1) – No (0) | 0.21 | 0.04 |  | | 0.00^C^ | | |  | | -0.28 | | | 0.69 |
|  | What is the highest level of schooling you have completed? Diploma: Yes (1) – No (0) | 0.16 | 0.04 |  | | 0.00^C^ | | |  | | -0.24 | | | 0.57 |
|  | In what sort of house do you live? Room rent: Yes (1) – No (0) | 0.09 | 0.04 |  | | 0.00^C^ | | |  | | -0.14 | | | 0.33 |
|  | What is your marriage status? Widow(er): Yes (1) – No (0) | -0.18 | -0.04 |  | | 0.00^C^ | | |  | | -0.64 | | | 0.28 |
|  | Human Dominance priming narrative group? Yes (1) – No (0) | -0.05 | -0.04 |  | | 0.00^C^ | | |  | | -0.20 | | | 0.09 |
|  | What is the highest level of schooling you have completed? Junior high: Yes (1) – No (0) | 0.48 | 0.03 |  | | 0.00^C^ | | |  | | -0.84 | | | 1.80 |
|  | Do you have children? Yes (1) – No (0) | 0.08 | 0.05 |  | | 0.00^C^ | | |  | | -0.15 | | | 0.30 |
|  | What is your gender? Female: Yes (1) – No (0) | -0.04 | -0.03 |  | | 0.00^C^ | | |  | | -0.17 | | | 0.09 |
|  | What is your gross household expenses per month? Five to 10 million: Yes (1) – No (0) | 0.06 | 0.03 |  | | 0.00^C^ | | |  | | -0.13 | | | 0.26 |
|  | What is your gross household expenses per month? 10 to 15 million: Yes (1) – No (0) | 0.16 | 0.03 |  | | 0.00^C^ | | |  | | -0.44 | | | 0.76 |
|  | What is your age? | 0.00 | 0.03 |  | | 0.00^C^ | | |  | | -0.01 | | | 0.01 |
|  | What is your gross household income per month? Refuse to answer: Yes (1) – No (0) | -0.04 | -0.02 |  | | 0.00^C^ | | |  | | -0.22 | | | 0.14 |
|  | Where is your current residence place? Urban area: Yes (1) – No (0) | 0.03 | 0.02 |  | | 0.00^C^ | | |  | | -0.11 | | | 0.17 |
|  | Do you have your own backyard? Yes (1) – No (0) | 0.03 | 0.02 |  | | 0.00^C^ | | |  | | -0.11 | | | 0.16 |
|  | What is your gross household income per month? About twice the average income in my country: Yes (1) – No (0) | 0.08 | 0.02 |  | | 0.00^C^ | | |  | | -0.38 | | | 0.54 |
|  | What is your gross household income per month? More than twice the average income in my country: Yes (1) – No (0) | 0.11 | 0.02 |  | | 0.00^C^ | | |  | | -0.52 | | | 0.73 |
|  | How often do you consume meat in a week? Two to three days a week: Yes (1) – No (0) | 0.03 | 0.02 |  | | 0.00^C^ | | |  | | -0.13 | | | 0.18 |

*p<.05; **p<.01; ^A^regression using enter method in a stepwise manner; ^B^regression using enter method; ^C^effect-size calculation using eta squared (F2); ^D^effect-size calculation using Hedge’s g; +small effect size F2>=0.02 (or in some cases of categorical dummy variable, using Cohen’s D/Hedges’g >= 0.2); ++medium effect size F2>=0.15 (or in some cases of categorical dummy variable, using cohen’s D/Hedges’g >=0.5); ^1^compared to respondents who fill the survey without having to read any narrative; ^2^compared to respondent who eat meat once a week.

Table 1 Main variables with demographic and other important determinants to GEA (continued)

|  | | GEA | | | | | Effect size | | | | | CI (95%) | | |
| --- | --- | --- | --- | --- | --- | --- | --- | --- | --- | --- | --- | --- | --- | --- |
|  |  | b | Std. b | | | |  | | | | | Lower | | Upper |
| Model 2^B^: Main variables with demographic and other determinants (continued) | | | | |  | | |  | |  | | |  |  |
|  | What is the highest level of schooling you have completed? Other: Yes (1) – No (0) | -0.15 | -0.01 |  | | 0.00^C^ | | |  | | -1.60 | | | 1.29 |
|  | What is the highest level of schooling you have completed? Bachelor: Yes (1) – No (0) | 0.02 | 0.01 |  | | 0.00^C^ | | |  | | -0.17 | | | 0.21 |
|  | How often do you visit a zoo or aquarium? Once a month: Yes (1) – No (0) | 0.03 | 0.01 |  | | 0.00^C^ | | |  | | -0.31 | | | 0.37 |
|  | How often do you visit a zoo or aquarium? Once a year: Yes (1) – No (0) | -0.01 | -0.01 |  | | 0.00^C^ | | |  | | -0.21 | | | 0.18 |
|  | Do you have pet? Yes (1) – No (0) | 0.01 | 0.01 |  | | 0.00^C^ | | |  | | -0.13 | | | 0.14 |

*p<.05; **p<.01; ^A^regression using enter method in a stepwise manner; ^B^regression using enter method; ^C^effect-size calculation using eta squared (F2); ^D^effect-size calculation using Hedge’s g; +small effect size F2>=0.02 (or in some cases of categorical dummy variable, using Cohen’s D/Hedges’g >= 0.2); ++medium effect size F2>=0.15 (or in some cases of categorical dummy variable, using cohen’s D/Hedges’g >=0.5); ^1^compared to respondents who fill the survey without having to read any narrative; ^2^compared to respondent who eat meat once a week.

Table 2 Main variables with demographic and other important determinants to AIS

|  | | AIS | | | Effect size | | CI (95%) | |
| --- | --- | --- | --- | --- | --- | --- | --- | --- |
|  |  | b | Std. b | |  | | Lower | Upper |
| Model 1^A^ : Main variables | |  |  |  |  |  |  |  |
| R = 0.339 | (Constant) | 3.18 |  | ** |  |  | 2.85 | 3.50 |
| R2 = 0.115 | Stewardship priming narrative group^1^? Yes (1) – No (0) | -0.14 | -0.11 | ** | 0.01^D^ |  | -0.24 | -0.04 |
| df =4, 652 | Human Dominance priming narrative group^1^? Yes (1) – No (0) | 0.07 | 0.06 |  | 0.00^C^ |  | -0.02 | 0.16 |
|  | ROS Personal | -0.22 | -0.22 | ** | 0.05^C^ | + | -0.29 | -0.15 |
|  | ROS Social | 0.12 | 0.23 | ** | 0.05^C^ | + | 0.08 | 0.16 |
| Model 2^B^: Main variables with demographic and other determinants | | | |  |  |  |  |  |
| R = 0.469 | (Constant) | 2.67 |  | ** |  |  | 2.10 | 3.25 |
| R2 = 0.22 | ROS Personal | -0.22 | -0.23 | ** | 0.05^C^ | + | -0.31 | -0.13 |
| df =39, 402 | ROS Social | 0.09 | 0.18 | ** | 0.03^C^ | + | 0.04 | 0.14 |
|  | How often do you consume meat in a week^2^? I don't consume meat: Yes (1) – No (0) | 0.35 | 0.17 | ** | 0.02^C^ | + | 0.15 | 0.54 |
|  | What is the highest level of schooling you have completed^3^? Diploma: Yes (1) – No (0) | 0.43 | 0.15 | ** | 0.02^C^ |  | 0.14 | 0.71 |
|  | In what sort of house do you live? Apartment^4^: Yes (1) – No (0) | 0.58 | 0.14 | ** | 0.01^C^ |  | 0.16 | 0.99 |
|  | What is the highest level of schooling you have completed^3^? Bachelor: Yes (1) – No (0) | 0.18 | 0.16 | ** | 0.01^C^ |  | 0.05 | 0.32 |
|  | What is your gross household expenses per month^5^? Above 25 million: Yes (1) – No (0) | -0.69 | -0.09 | * | 0.01^C^ |  | -1.35 | -0.02 |
|  | What is your gross household income per month? More than twice the average income in my country: Yes (1) – No (0) | 0.41 | 0.09 |  | 0.01^C^ |  | -0.03 | 0.86 |
|  | What is your age? | 0.01 | 0.11 |  | 0.01^C^ |  | -0.00 | 0.01 |
|  | In what sort of house do you live? Own house: Yes (1) – No (0) | 0.10 | 0.10 |  | 0.01^C^ |  | -0.02 | 0.21 |
|  | Where is your current residence place? Urban area: Yes (1) – No (0) | -0.09 | -0.08 |  | 0.01^C^ |  | -0.19 | 0.02 |
|  | What is the highest level of schooling you have completed? Other: Yes (1) – No (0) | -0.79 | -0.08 |  | 0.00^C^ |  | -1.82 | 0.24 |
|  | Is religion important for you? Yes (1) – No (0) | 0.25 | 0.07 |  | 0.00^C^ |  | -0.10 | 0.59 |
|  | How often do you visit a zoo or aquarium? Once a month: Yes (1) – No (0) | 0.16 | 0.07 |  | 0.00^C^ |  | -0.08 | 0.40 |
|  | What is your gender? Female: Yes (1) – No (0) | -0.06 | -0.06 |  | 0.00^C^ |  | -0.15 | 0.03 |
|  | Stewardship priming narrative group? Yes (1) – No (0) | -0.07 | -0.05 |  | 0.00^C^ |  | -0.19 | 0.05 |
|  | Do you have children? Yes (1) – No (0) | 0.08 | 0.07 |  | 0.00^C^ |  | -0.08 | 0.24 |
|  | What is your marriage status? Married: Yes (1) – No (0) | -0.08 | -0.07 |  | 0.00^C^ |  | -0.27 | 0.10 |

*p<.05; **p<.01; ^A^regression using enter method in a stepwise manner; ^B^regression using enter method; ^C^effect-size calculation using eta squared (F2); ^D^effect-size calculation using Hedge’s g; +small effect size F2>=0.02 (or in some cases of categorical dummy variable, using Cohen’s D/Hedges’g >= 0.2); ++medium effect size F2>=0.15 (or in some cases of categorical dummy variable, using cohen’s D/Hedges’g >=0.5); ^1^compared to respondents who fill the survey without having to read any narrative; ^2^compared to respondent who eat meat once a week; ^3^compared to respondent with master/PhD; ^4^compared to respondent who is still live with their parents; ^5^compared to those respondents whose expenses is below IDR five millions a month.

Table 2 Main variables with demographic and other important determinants to AIS (continued)

|  | | AIS | | | Effect size | | CI (95%) | |
| --- | --- | --- | --- | --- | --- | --- | --- | --- |
|  |  | b | Std. b | |  | | Lower | Upper |
| Model 2^B^: Main variables with demographic and other determinants (continued) | | | |  |  |  |  |  |
|  | How often do you visit a zoo or aquarium? Once every six month: Yes (1) – No (0) | -0.08 | -0.04 |  | 0.00^C^ |  | -0.28 | 0.11 |
|  | How often do you visit a zoo or aquarium? Once every two or more year: Yes (1) – No (0) | -0.05 | -0.05 |  | 0.00^C^ |  | -0.17 | 0.07 |
|  | How often do you consume meat in a week? Four to six days a week: Yes (1) – No (0) | 0.05 | 0.03 |  | 0.00^C^ |  | -0.10 | 0.20 |
|  | What is your gross household income per month? About the average income in my country: Yes (1) – No (0) | -0.04 | -0.04 |  | 0.00^C^ |  | -0.18 | 0.09 |
|  | What is your gross household income per month? About twice the average income in my country: Yes (1) – No (0) | -0.09 | -0.03 |  | 0.00^C^ |  | -0.42 | 0.24 |
|  | What is the highest level of schooling you have completed? Junior high: Yes (1) – No (0) | 0.24 | 0.02 |  | 0.00^C^ |  | -0.70 | 1.19 |
|  | What is your gross household income per month? About the minimum income in my country: Yes (1) – No (0) | -0.03 | -0.02 |  | 0.00^C^ |  | -0.18 | 0.11 |
|  | In what sort of house do you live? Room rent: Yes (1) – No (0) | 0.04 | 0.03 |  | 0.00^C^ |  | -0.13 | 0.21 |
|  | What is your gross household expenses per month? Five to 10 million: Yes (1) – No (0) | 0.03 | 0.02 |  | 0.00^C^ |  | -0.11 | 0.17 |
|  | How often do you consume meat in a week? Everyday: Yes (1) – No (0) | -0.03 | -0.02 |  | 0.00^C^ |  | -0.19 | 0.12 |
|  | What is your marriage status? Widow(er): Yes (1) – No (0) | -0.06 | -0.02 |  | 0.00^C^ |  | -0.38 | 0.27 |
|  | Do you have pet? Yes (1) – No (0) | -0.01 | -0.01 |  | 0.00^C^ |  | -0.11 | 0.08 |
|  | Do you have your own backyard? Yes (1) – No (0) | 0.01 | 0.01 |  | 0.00^C^ |  | -0.08 | 0.11 |
|  | What is the highest level of schooling you have completed? Senior high: Yes (1) – No (0) | 0.02 | 0.01 |  | 0.00^C^ |  | -0.19 | 0.24 |
|  | What is your gross household expenses per month? Refuse to answer: Yes (1) – No (0) | 0.01 | 0.01 |  | 0.00^C^ |  | -0.12 | 0.14 |
|  | Human Dominance priming narrative group? Yes (1) – No (0) | 0.00 | 0.00 |  | 0.00^C^ |  | -0.10 | 0.11 |
|  | What is your gross household expenses per month? 10 to 15 million: Yes (1) – No (0) | -0.02 | -0.00 |  | 0.00^C^ |  | -0.45 | 0.41 |

*p<.05; **p<.01; ^A^regression using enter method in a stepwise manner; ^B^regression using enter method; ^C^effect-size calculation using eta squared (F2); ^D^effect-size calculation using Hedge’s g; +small effect size F2>=0.02 (or in some cases of categorical dummy variable, using Cohen’s D/Hedges’g >= 0.2); ++medium effect size F2>=0.15 (or in some cases of categorical dummy variable, using cohen’s D/Hedges’g >=0.5); ^1^compared to respondents who fill the survey without having to read any narrative; ^2^compared to respondent who eat meat once a week; ^3^compared to respondent with master/PhD; ^4^compared to respondent who is still live with their parents; ^5^compared to those respondents whose expenses is below IDR five millions a month.

Table 2 Main variables with demographic and other important determinants to AIS (continued)

|  | | AIS | | | Effect size | | CI (95%) | |
| --- | --- | --- | --- | --- | --- | --- | --- | --- |
|  |  | b | Std. b | |  | | Lower | Upper |
| Model 2^B^: Main variables with demographic and other determinants (continued) | | | |  |  |  |  |  |
|  | How often do you visit a zoo or aquarium? Once a year: Yes (1) – No (0) | -0.01 | -0.00 |  | 0.00^C^ |  | -0.15 | 0.14 |
|  | What is your gross household income per month? Refuse to answer: Yes (1) – No (0) | 0.00 | 0.00 |  | 0.00^C^ |  | -0.13 | 0.13 |
|  | Do you have any affiliation to religious organization? Yes (1) – No (0) | 0.00 | 0.00 |  | 0.00^C^ |  | -0.10 | 0.10 |
|  | How often do you consume meat in a week? Two to three days a week: Yes (1) – No (0) | 0.00 | 0.00 |  | 0.00^C^ |  | -0.11 | 0.11 |

*p<.05; **p<.01; ^A^regression using enter method in a stepwise manner; ^B^regression using enter method; ^C^effect-size calculation using eta squared (F2); ^D^effect-size calculation using Hedge’s g; +small effect size F2>=0.02 (or in some cases of categorical dummy variable, using Cohen’s D/Hedges’g >= 0.2); ++medium effect size F2>=0.15 (or in some cases of categorical dummy variable, using cohen’s D/Hedges’g >=0.5); ^1^compared to respondents who fill the survey without having to read any narrative; ^2^compared to respondent who eat meat once a week; ^3^compared to respondent with master/PhD; ^4^compared to respondent who is still live with their parents; ^5^compared to those respondents whose expenses is below IDR five millions a month.
