# Supplementary material for: The role of religious narratives and religious orientation towards concerns for the natural environment and animal welfare
Source: PLoS One. 2022 Aug 11;17(8):e0271515. doi: 10.1371/journal.pone.0271515 (PMC9371258; doi:10.1371/journal.pone.0271515)
Supplement: Attachment — Submitted filename: Response to Reviewer.docx [file pone.0271515.s009.docx]

**Response to reviewers**

Dear Reviewers,

Thank you for your review and comments on our manuscript addressing critical points in order for us to make it better and more concise. We are very glad to have this review and honored that you provide your valuable time for reviewing this in such a swift manner.

In the tables below we indicated how we revised our manuscript in detail.

Thank you very much in advance for your consideration.

With kind regards,

Dexon Pasaribu, Pim Martens and Bagus Takwin

| 1 | Is the manuscript presented in an intelligible fashion and written in standard English?  There are some issues with grammar and expression in English throughout the paper that need attention. Some ideas need to be expressed more clearly. However, this is a very interesting piece of research and I encourage you to make the small changes necessary for publication |
| --- | --- |
|  | Response:  Dear Esteemed Reviewer,  we sincerely grateful to your feedback regarding the English quality of our paper. We have addressed this issue and have made some improvement on the quality of the written English for academic paper. |

| 2 | Line 295. Numbers do not add up. If 276 participants are excluded from a total of 929, then N=653, not 657. See also 464-484 |
| --- | --- |
|  | Response:  Dear Esteemed Reviewer,  thank you for mentioning this mistake. Yes indeed we made a mistake. We revised the manuscript to “272 participants removed” (not 276). |

| 3 | 353-379 I could not understand the description of the scales here. Is this a 22 item scale of 12 + 10 items, and is it separate from the 9-item GAE scale? |
| --- | --- |
|  | Response:  Dear esteemed reviewer,  thank you for addressing about the unclear description for the Thompson and Barton’s EASE scale. We reformulated the paragraph to better describe this scale:  In the fourth section, Thompson & Barton's [4] Ecocentric-Anthropocentric Scale of Environmental Attitude (EASEA) is used to measure environmental attitudes which consists of three dimensions. Amérigo et al. [6] argue that ecocentrism seems to include two concepts: the self in nature (egobiosphere) and nature itself (biosphere). In ecocentrism motives, on the one hand, there are items about physical or psychological benefits for the individual, brought about by the mere fact of being in or thinking about nature (e.g. “Being out in nature is a great stress reducer for me”). These are related to the positive emotional effects produced by contact with nature where the protagonist is the self and it is the only direct beneficiary of the goodness of the natural environment which could be considered to be related to an egoistic dimension [6]. On the other hand, the remaining ecocentric items refer to biospheric aspects that emphasize the intrinsic value of Nature (e.g. “Nature is valuable for its own sake”) which may be oriented into two different viewpoints of (a) a psychosocial perspective that contemplates the human-being-in-nature and in which the environment is valued as an element that procures the individual’s physical and psychological well- being, and (b) a strictly biospheric dimension in which the environment is valued intrinsically and that contemplates the nonhuman elements of nature [6]. In short, Amérigo et al. [6] propose that there are anthropocentric valuation in the Thompson & Barton’s [4] ecocentric scale. Thus it is very difficult to differentiate whether environmental concerns stem from ecocentric or anthropocentric motives or both. Therefore, this study only use the general environmental apathy scale to measure whether a person has apathy disposition towards their natural environment.  From total 31 items of Thompson & Barton's [4] Ecocentric-Anthropocentric Scale of Environmental Attitude (EASEA), tThere are total of 12 questions measuring ecocentric motive where nature is valued for its own sake, and therefore, judged that it deserves protection because of its intrinsic value. There are total of 10 questions measuring anthropocentric motive where the natural environment is valued due to its importance in maintaining or enhancing the quality of life for humankind and therefore should be protected [4] (p. 149). And lastly, There are total of nine questions measuring general apathy toward the environment. As explained above, this study only use the general environment apathy (GEA) scale. Type of statements that are being asked to rate were, for example, ‘environmental threats such as deforestation and ozone depletion have been exaggerated,’ too much emphasis has been placed on conservation,’ or ‘I don't care about environmental problems.’ However, after principal axis factoring factor analysis (S4 Missing case analysis, Factor Analysis and Reliability; Table 10-18), this study reduced the items to only seven items, and found that GEA consists of two factors instead of one. However, after confirming a good Cronbach alpha’s reliability in one factor model, the present study decided to retain the environmental apathy dimension as it was originally, a one factor construct (model two, see Table 2) |

| 4 | 357 has 10 items in the anthropocentric motive measure, but there are only 9 questions in 383. Is this right? |
| --- | --- |
|  | Response:  Dear Esteemed Reviewer,  we want to thank you for your correction. Indeed, we were mistaken. We wrote,  “In the general environment apathy scale, the type of statement being asked are, for example, ‘environmental threats such as deforestation and ozone depletion have been exaggerated,’ too much emphasis has been placed on conservation,’ or ‘I don't care about environmental problems.’ There are total of **nine questions in the anthropocentric motive dimension**. However, after principal axis factoring factor analysis (S4 Missing case analysis, Factor Analysis and Reliability; Table 10-18), this study not only reduced the items to only seven items..”  And have corrected it to  “From total 31 items of Thompson & Barton's [4] Ecocentric-Anthropocentric Scale of Environmental Attitude (EASEA), there are total of 12 questions measuring ecocentric motive where nature is valued for its own sake, and therefore, judged that it deserves protection because of its intrinsic value. There are total of 10 questions measuring anthropocentric motive where the natural environment is valued due to its importance in maintaining or enhancing the quality of life for humankind and therefore should be protected [4] (p. 149). And lastly, **There are total of nine questions measuring general apathy toward the environment.** As explained above, this study only use the general environment apathy (GEA) scale. Type of statements that are being asked to rate were, for example, ‘environmental threats such as deforestation and ozone depletion have been exaggerated,’ too much emphasis has been placed on conservation,’ or ‘I don't care about environmental problems.’ However, after principal axis factoring factor analysis (S4 Missing case analysis, Factor Analysis and Reliability; Table 10-18), this study reduced the items to only seven items |

| 5 | 392 Where does the AIS come from? |
| --- | --- |
|  | Response:  Dear Esteemed Reviewer,  thank you for this question. AIS score is the mean from all 31 items (consist of 4 items from animal use issue, 4 items from animal integrity destruction issue, 7 item from killing animal and animal welfare deprivation issue, 3 items from animal experimentation issue, 4 items from animal genetic change issue, 4 items from harming animals for environmental reasons issue and lastly, harming animals for social reasons issue). We add additional sentence to the paragraph:  “..Using principal axis factoring factor analysis (S4 Missing case analysis, Factor Analysis and Reliability; Table 3-5), the original ‘killing animal’ and ‘animal deprivation’ issues were identified as one factor (Table 3). Thus, the present study reduced AIS to only 31 items, conveyed only 7 factors. Overall AIS score is the mean score from all 31 items.” |

| 6 | 448-462 The conclusions from the mean scores are not justifiable. For example, "The mean score for ES was 2.82 (SD=1.01) indicating that, on the whole, the respondents have above average disposition towards viewing their religious practices as an instrument for social affiliation." This is not a valid conclusion, unless you have evidence that the mean score for a randomly selected sample of the population scores on average lower than 2.82. |
| --- | --- |
|  | Response:  Dear Esteemed Reviewer,  thank you for your feedback about translating mean score in this section. We have revised the paragraph:  “The mean score for IP was 4.23 (SD=0.53, with maximum score of five) indicating that, overall, the respondents mostly expresses strong agreement to items that indicate intrinsic motivation and commitment to their personal religious life. The mean score for ES was 2.82 (SD=1.01) indicating that, the respondents tend to be undecided to questionnaire statement that indicates religious practices as an instrument for social affiliation. The general environmental apathy mean score was 2.55 (SD = 0.72), indicating that, the respondents mostly express disagreement or neutrality to item that indicate environmental apathy. The mean score of overall acceptability toward harming animal (AIS) was 2.57 (SD=0.53), indicating that, in general, respondents find that statements about harming animals are unacceptable for them. Except for the issues of animal use (mean of 3.13, SD=0.66), animal experimentation (mean of 3.01, SD=0.85) and genetic change (mean of 3.3, SD=0.74), the remaining subscales of animal integrity destruction (mean of 2.43, SD=0.80), killing-welfare deprivation of animal (mean of 2.12, SD=0.78), harm (animals) for environmental issue (mean of 2.40, SD=0.80), and harm (animals) for social issue (mean of 2.15, SD=0.78) most of the respondents answered lower acceptability for harming animals.” |
